# Ejection fraction in myocardial perfusion imaging assessed with a dynamic phantom: comparison between IQ-SPECT and LEHR

**DOI:** 10.1186/s40658-017-0187-2

**Published:** 2017-07-19

**Authors:** Eero Hippeläinen, Teemu Mäkelä, Touko Kaasalainen, Erna Kaleva

**Affiliations:** 1 HUS Medical Imaging Center, Clinical Physiology and Nuclear Medicine, Helsinki University Hospital, University of Helsinki, POB 340, FI-00029 HUS Helsinki, Finland; 2 Department of Physics, University of Helsinki, Helsinki, Finland; 3 HUS Medical Imaging Center, Radiology, Helsinki University Hospital, University of Helsinki, Helsinki, Finland; 4 Radiation Therapy Unit of Lahti, Tampere University Hospital, Lahti, Finland

**Keywords:** Myocardial perfusion imaging, SPECT, Gated CT, IQ-SPECT, LEHR

## Abstract

**Background:**

Developments in single photon emission tomography instrumentation and reconstruction methods present a potential for decreasing acquisition times. One of such recent options for myocardial perfusion imaging (MPI) is IQ-SPECT. This study was motivated by the inconsistency in the reported ejection fraction (EF) and left ventricular (LV) volume results between IQ-SPECT and more conventional low-energy high-resolution (LEHR) collimation protocols. IQ-SPECT and LEHR quantitative results were compared while the equivalent number of iterations (EI) was varied. The end-diastolic (EDV) and end-systolic volumes (ESV) and the derived EF values were investigated.

A dynamic heart phantom was used to produce repeatable ESVs, EDVs and EFs. Phantom performance was verified by comparing the set EF values to those measured from a gated multi-slice X-ray computed tomography (CT) scan (EF_True_). The phantom with an EF setting of 45, 55, 65 and 70% was imaged with both IQ-SPECT and LEHR protocols. The data were reconstructed with different EI, and two commonly used clinical myocardium delineation software were used to evaluate the LV volumes.

**Results:**

The CT verification showed that the phantom EF settings were repeatable and accurate with the EF_True_ being within 1% point from the manufacture’s nominal value. Depending on EI both MPI protocols can be made to produce correct EF estimates, but IQ-SPECT protocol produced on average 41 and 42% smaller EDV and ESV when compared to the phantom’s volumes, while LEHR protocol underestimated volumes by 24 and 21%, respectively. The volume results were largely similar between the delineation methods used.

**Conclusions:**

The reconstruction parameters can greatly affect the volume estimates obtained from perfusion studies. IQ-SPECT produces systematically smaller LV volumes than the conventional LEHR MPI protocol. The volume estimates are also software dependent.

## Background

Myocardial perfusion imaging (MPI) using single photon emission computed tomography (SPECT) has been shown to have good prognostic power for diagnosing coronary artery disease [1, 2]. Gated MPI has shown to significantly reduce the number of so-called borderline cases when interpreted simultaneously with stress and rest perfusion images [3]. In addition, post-stress left ventricle ejection fraction (LVEF) and end-systolic volume (ESV) have incremental prognostic value over perfusion information in predicting cardiac death [4].

In recent years, developments in both SPECT MPI reconstruction methods and hardware have been in part affected by clinical desire of increased and more economical patient throughput. In addition to novel SPECT camera models dedicated to MPI [5], introduction of collimator-detector response compensation and other advanced noise reduction methods into the iterative reconstruction algorithms [6] have made faster or ‘low activity’ MPI imaging generally available [7].

IQ-SPECT is a cardiac imaging software and hardware option for Siemens general purpose gamma cameras [8] (Siemens Healthineers, Erlangen, Germany). The three main components of IQ-SPECT are variable-focus collimators (SmartZoom), a cardio-centric acquisition orbit and an iterative reconstruction algorithm incorporating collimator-detector response compensation for the SmartZoom collimators [9]. It has been shown using static cardiac phantoms that IQ-SPECT preserves both image quality and the results of quantitative measurements with a quarter of the acquisition time or a quarter of administered activity used in conventional MPI [8, 10]. Caobelli et al. have also compared the LEHR protocol with a one-fourth acquisition time IQ-SPECT protocol and found no significant differences in perfusion scores [11]. Recently, they have shown with patient data that one-eighth time IQ-SPECT protocol produces statistically comparable results with the one-fourth protocol and could provide significant improvement in patient throughput of an MPI centre [12]. However, Havel et al. have shown how IQ-SPECT produces significantly higher summed stress, rest and difference scores, lower EF values and higher left ventricular (LV) volumes than the conventional LEHR imaging protocol with filtered back projection reconstruction [13]. On the other hand, Horiguchi et al. have shown that the number of projections in an IQ-SPECT study has a significant effect on thallium-201 MPI results [14]. By increasing the number of projections per camera head from the recommended 17 to 36, they produced MPI images equivalent to those using conventional SPECT. In addition to reconstruction methods and imaging protocol, it has also been shown by Caobelli et al. that the positioning process of the cardio-centric orbit at the beginning of the acquisition can have an effect on diagnostic accuracy of the IQ-SPECT imaging [15]. As the published results remain inconsistent, it is difficult to have a clear conclusion on the reliability of IQ-SPECT.

In a recent study, Joergensen and Hansson measured 28 patients’ EFs using IQ-SPECT from two different reconstruction datasets and also with multi-gated planar equilibrium radionuclide ventriculography (MUGA) [16]. The reconstruction parameters for IQ-SPECT were 15 iterations, 2 subsets and 10-mm Gaussian post-filter (protocol A) and 12 iterations, 1 subset and 10-mm Gaussian post-filter (protocol B). They concluded that none of the IQ-SPECT protocols evaluated in the study were comparable with MUGA, and they stated that ‘[IQ-SPECT protocol A or B] is not suitable for evaluation of LVEF [(left ventricular ejection fraction)] in critical settings, e.g., in control of chemotherapy or evaluation of cardiac pumping efficiency’.

In this study, we validate a dynamic heart phantom using gated CT imaging. Then, we evaluate the absolute LV volume quantification accuracy of IQ-SPECT and compare the calculated LVEF to a conventional LEHR acquisition protocol (EF_IQSPECT_ and EF_LEHR_, respectively). Our study is motivated by the inconsistency in the EF and LV volume results between IQ-SPECT and LEHR protocols, as discussed above. In addition, to our knowledge, the effect of reconstruction parameters on EF has not been explicitly studied in any prior IQ-SPECT investigations. We investigate how the number of reconstruction iterations affects the EF estimation in a high signal, no background phantom setup.

## Methods

EF_IQSPECT_ and EF_LEHR_ were measured using a dynamic heart phantom (PTW-Freiburg GmbH, Freiburg, Germany) capable of varying EF and beats per minute (BPM). The phantom can also give a trigger signal to a SPECT or computed tomography (CT) system via an electrocardiography (ECG) connector.

The EF [%] was calculated from the measured volumes using the equation

1 $$ \mathrm{E}\mathrm{F}=\frac{\left(\mathrm{EDV}-\mathrm{ESV}\right)}{\mathrm{EDV}}\times 100\% $$

where EDV and ESV are the largest and smallest phantom volumes corresponding to the end-diastolic and end-systolic volumes, respectively. The heart phantom volumes were validated using gated CT imaging (EDV_True_, ESV_True_ and EF_True_) as a reference. These were then compared with estimates from clinical IQ-SPECT and LEHR acquisition protocols.

### Dynamic heart phantom

**Fig. 1:**
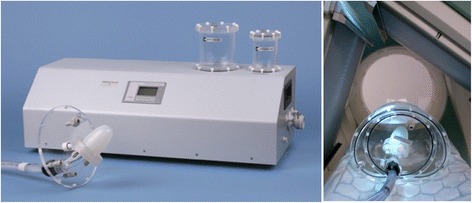
The dynamic heart phantom. *Left*—the membrane and pump units. *Right*—the phantom assembled and ready for measurement in the gamma camera. The membrane unit installed is shown inside a NEMA IEC PET body phantom and positioned for IQ-SPECT acquisition

The phantom consists of two main parts: a pump unit and a membrane unit. The membrane unit of the phantom was placed within a NEMA IEC PET body phantom (Fig. 1). The EDV is varied by changing the amount of water pumped into the membrane unit, while ESV remains constant. The nominal EF settings used in this study were 45, 55, 65 and 70%. According to the manufacturer, the phantom pump unit produces highly accurate and repeatable volumes and thus possible error between cardiac cycles was considered negligible. However, the membrane sizes may vary slightly between units, and thus, the ESV was verified with a CT scan and manual measurements.

During the IQ-SPECT and LEHR acquisitions, the myocardial volume of the phantom was filled with a solution of 300 MBq of ^99m^Tc-pertechnetate diluted with 400 ml of distilled water. A high amount of ^99m^Tc activity was used to produce statistically high-quality SPECT projection data. This non-clinical concentration was used to distinguish the effect of iteration numbers from other confounding factors such as (denoising) filter choice or delineation software performance on low statistics data. The activity concentration within the myocardium space was verified with gamma counter measurements to ensure consistency between acquisitions. The CT was carried out without introducing radionuclides or contrast agents.

### Phantom validation with gated CT

To validate phantom volume accuracy and consistency, the dynamic heart phantom was scanned with a second-generation dual-source Siemens SOMATOM Definition Flash CT system. ECG-triggered cardiac CT scans were performed at 60 bpm with four different EF settings: 45, 55, 65 and 70%. EF 65% was additionally scanned at heart rates of 40, 50, 60 and 70 bpm. The scanning parameters used in the measurements were tube voltage 100 kVp, slice collimation 2 × 128 × 0.6 mm with z-flying focal spot, gantry rotation time 280 ms, quality reference tube current-time product 320 mAs per rotation (CareDose 4D), and pitch factor 3.4. The time resolution of the scans was 75 ms. The mean volume CT dose index and dose length product were 36 mGy and 570 mGy cm, respectively. For comprehensive evaluation of the phantom volumes, images with a slice thickness of 0.6 mm and an increment of 0.6 mm were reconstructed with an I26f reconstruction kernel (SAFIRE level 2) from 21 cardiac phases (0–100% with 5% intervals) for each scan. The reconstructed image field of view was set to 150 mm and the matrix size to 256 × 256 resulting in near-isotropic voxels.

For ESV validation, a non-gated CT scan of the resting state phantom was performed with a chest CT scan protocol. The scanning parameters were 120 kVp, 128 × 0.6 mm slice collimation, 0.5-s rotation time, 0.6 pitch and 120 quality reference mAs. Images with a slice thickness of 0.6 mm, an increment of 0.6 mm, 150 mm FOV and 512 × 512 image matrix were reconstructed using the B30f kernel. In addition, ESV was manually measured by dipping a relaxed inner membrane unit into a water path. The water bath was placed on a high precision scale, and the replaced water mass was measured with the scale and interpreted as volume.

### Gated CT image analysis

**Fig. 2:**
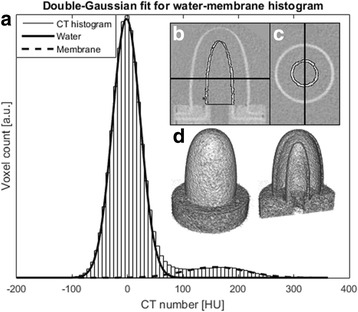
CT imaging was used in the phantom validation **d**. Segmentation between phantom wall and water was performed by fitting a double Gaussian function to the CT volume histogram **a**. The volume corresponding to the ESV is delineated in **b** and **c** by the *thin black line* including the inner membrane. The *thick black line* indicates the corresponding slice locations in **b** and **c**. The segmentation was verified by visually viewing the axial slices **c**

The following analysis was carried out individually on all the data sets and cardiac phases and on the non-gated reference volume. Each volume was resampled to align along the horizontal long axis with isotropic 0.6-mm resolution using bicubic interpolation. A double-peaked Gaussian function (see Fig. 2) was fitted to the volume histogram using a simplex search method of Lagarias et al. [17] implemented in MATLAB (MathWorks, Natick, MA, USA). The water-membrane segmentation decision threshold was set at the half-value of the obtained Gaussians’ peak locations. As the CT number of water is expected to be near zero, the resulting threshold is approximately half of the mean CT number of the membrane wall. While preserving the membrane boundary 3-D morphological dilation, erosion and hole-filling were applied to remove the clutter caused by image noise. Each segmented slice was verified visually to only hold voxels belonging to the innermost phantom volume and the corresponding membrane wall. LV volumes from all the 4-D acquisitions and time points were determined to ensure segmentation algorithm stability. The EF_True_ values for each phantom setting were calculated using the smallest (ESV_True_) and largest volumes (EDV_True_). EF 65% acquisitions with different BPM settings were used to further assess the phantom measurement and analysis consistency and repeatability.

### IQ-SPECT and LEHR acquisitions

#### Acquisition protocols

Both acquisitions were performed using the same dual head gamma camera (Siemens Symbia T2) using ECG-gating with eight bins. The data were acquired into a 128 × 128 matrix producing a 4.8-mm voxel size. The energy window was centred at 140 keV with 15% width. The IQ-SPECT protocol was based on the manufacturer’s recommendations with an extended acquisition time of 30 s per view. IQ-SPECT covers a 204° scanning arc with an angular step of 6.12°. A total of 34 projections were acquired. To ensure repeatable positioning, the phantom was first aligned with camera’s lasers. Then, the first cardio-centric orbit setup was marked on the surface of the phantom with a cobalt-57 pen-point and a regular marker. The following cardio-centric orbit setups were focused with the help of the cobalt-57 pen-point marker on the surface markings of the phantom. The radius of the rotation was constant (28 cm) for the IQ-SPECT due to the cardio-centric orbit.

The LEHR protocol had a scanning arc of 180° with an angular step of 1.4° and an acquisition time of 25 s per view. A total of 128 projections were acquired with camera heads in the 90° cardiac configuration. For the LEHR acquisitions, the phantom was positioned at the centre of the field of view (FOV), but the acquisition was carried out using a non-circular orbit and auto-contouring as is routinely carried out in clinical scanning.

#### Image reconstruction and data analysis

The gated LEHR projection data were reconstructed using ordered subset expectation maximization (OSEM)-based Flash3D iterative reconstruction (Siemens Healthineers, Erlangen, Germany). IQ-SPECT data were reconstructed using Siemens implementation of conjugate-gradient iterative algorithm. No attenuation correction was used as it was not supported in the reconstruction software for gated data. Because the algorithms included a collimator-detector response compensation and the projection data had high signal-to-noise ratio, filtering was not applied. The images were reconstructed using different combinations of numbers of iterations and subsets, but henceforth, the results will be presented as a function of equivalent number of iterations (EI), i.e. the product of the number of iterations and the number of subsets.

The EF and LV volumes were calculated using Quantitative Gated SPECT software (QGS, Cedars-Sinai Medical Center, Los Angeles, USA) and Corridor4DM (4DM, University of Michigan Medical Center, USA) with an automatic myocardium delineation. For 4DM, ‘Mid membraneous Septum’ algorithm was used for delineation [18].

## Results

### Phantom validation

**Fig. 3:**
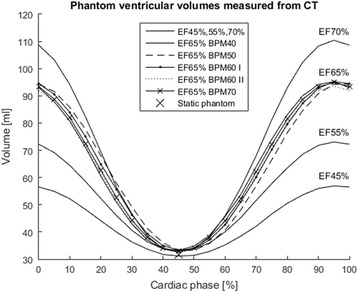
The phantom volumes analysed from the gated CT data. The largest and smallest volume values were used to calculate the EF. All the shown 21 phase volumes obtained from the gated CT scans were analysed as an additional verification of the phantom and CT analysis method: the expected sinusoidal-like curves were produced for all the settings. The values include the innermost water volume and the phantom membrane surrounding it as shown in Fig. 2b, c

The LV volumes of different cardiac phases measured from the gated CT are shown in Fig. 3. The measured ESV_True_, EDV_True_ and EF_True_ values are presented in Table 1. The differences between measured EFs and phantom settings were less than one percentage point. In addition, the ESV was verified with non-gated CT scan and with manual measurements, and the ESV_True_ was found to be 34.0 and 32.9 ml, respectively.

**Table 1:** ESV_True_, EDV_True_ and EF_True_ measured from gated CT images

Phantom settings	bpm	EDV_True_ [ml]	ESV_True_ [ml]	EDV-ESV [ml]	EF_True_ [%]
EF 45%	60	56.9	31.2	25.8	45.3
EF 55%	60	73.1	33.1	40.0	54.8
EF 65%	40	94.3	33.2	61.2	64.8
EF 65%	50	95.2	33.3	61.9	65.0
EF 65% I	60	93.6	32.6	61.0	65.2
EF 65% II	60	94.9	32.9	62.0	65.1
EF 65%	70	95.2	33.2	61.9	65.1
EF 70%	60	110.4	32.5	77.9	70.6

### IQ-SPECT and LEHR

**Fig. 4:**
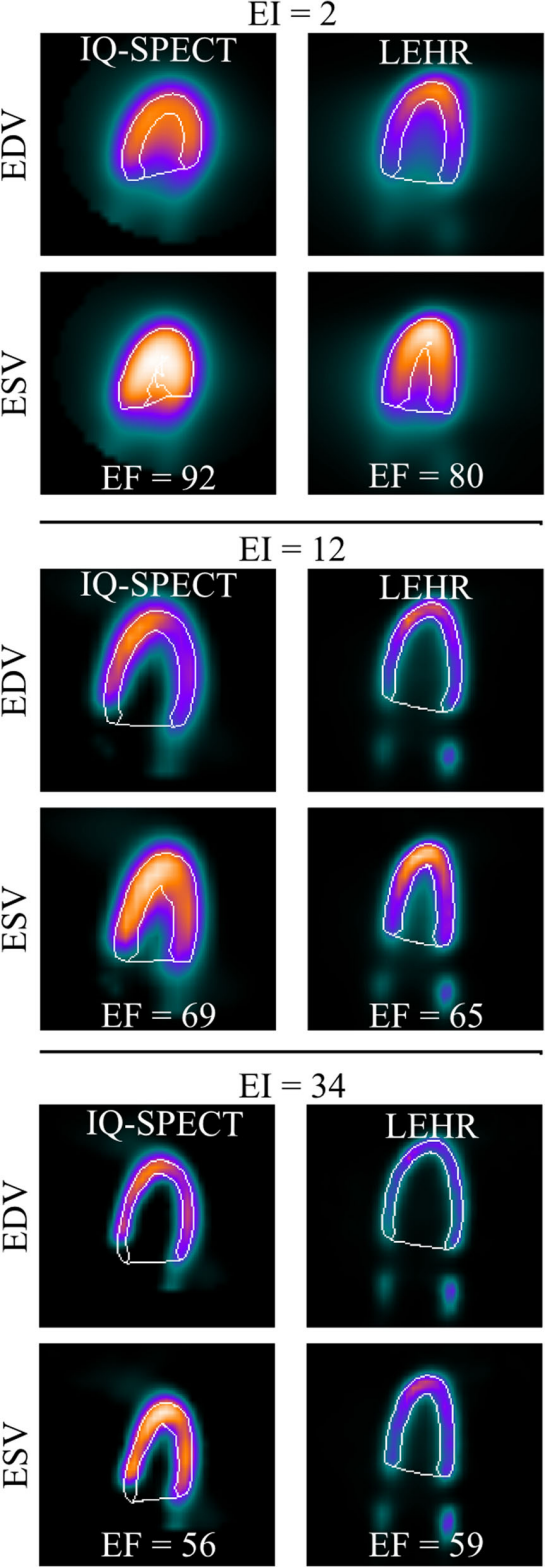
SPECT images of the phantom. Representative slices of the phantom EF setting 65% acquired using LEHR and IQ-SPECT protocols. Images are at end-diastolic (EDV) and end-systolic (ESV) phases after 2, 12 and 34 equivalent iterations presented with corresponding EF values. The contours present QPS software volume delineations

Representative gated SPECT images for EF setting 65% with different EI values are shown in Fig. 4. There are distinct differences between LEHR and IQ-SPECT images, resulting in smaller ventricle volumes in the latter. For example the ‘myocardium’ is shorter and thicker in the IQ-SPECT than in LEHR images. Also, the apex thickening, often seen in clinical images, is visible in the IQ-SPECT data.

**Fig. 5:**
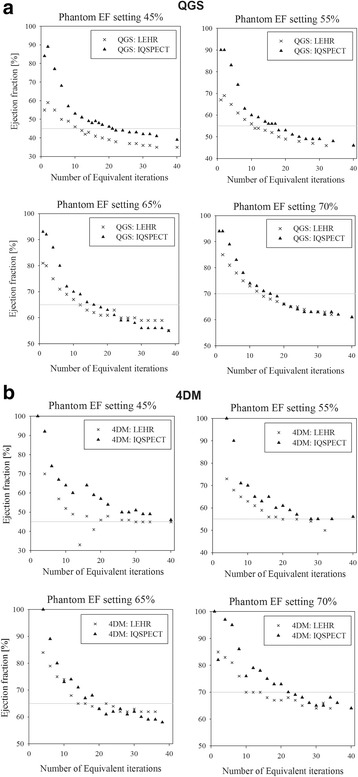
EF_LEHR_ and EF_IQSPECT_ estimates as a function of number of reconstruction equivalent iterations for the dynamic heart phantom, analysed using **a** QGS and **b** 4DM software. The phantom was imaged with 45, 55, 65 and 70% EF settings (*grey solid line*)

**Fig. 6:**
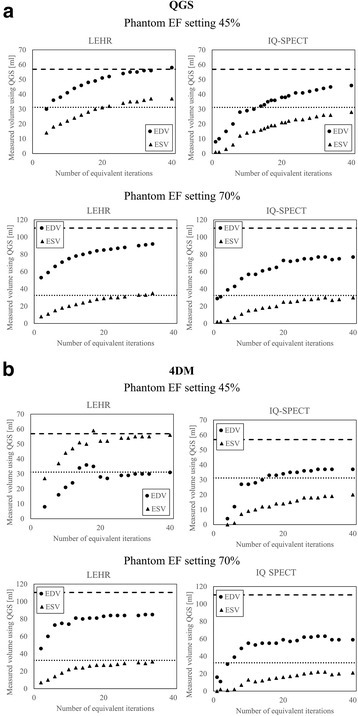
ESV and EDV estimates of the dynamic heart phantom using LEHR and IQ-SPECT protocols and analysed using **a** QGS and **b** 4DM. The phantom results for phantom EF settings 45 and 70% are shown. The *long and short dashed lines* represent the EDV_true_ and the ESV_true_ volumes, respectively, determined from the gated CT images

The EF values for the IQ-SPECT and LEHR protocols as a function of EI are shown in Fig. 5a, b. In both protocols, the EF calculations depend highly on the number of reconstruction iterations; for low number of iterations, both algorithms greatly overestimate the EF. This results from the slow convergence of the reconstruction algorithms and underestimations of the volumes (also seen in Fig. 6). Both algorithms slowly approach a stable EF value around 30 EIs, but with higher EI, proceed to underestimate EF from 5 to 10 percentage points.

The volume estimates of ESV and EDV for different number of EIs are shown in Fig. 6 for both imaging protocols with corresponding true volumes (dashed lines) determined during CT validation. EDVs and ESVs can be seen to converge towards the true physical volumes produced by the phantom. The volume estimates keep increasing over the studied EI range, but the ‘stroke volume’ (EDV-ESV) reaches a constant after approximately 10 iterations. This explains the decreasing phenomenon in the calculated EF with QGS software (Fig. 5a); the EDV and ESV increase, but the difference between them remains constant. In contrast, 4DM does not alter the cardiac muscle thickness as much as QGS with higher EIs. Therefore, similar increasing volume phenomenon is not as clearly present (Fig. 5b), and EF is more stable with high number of EIs. However, The IQ-SPECT produced systematically smaller volumes than LEHR protocol at the studied iterations range.

**Fig. 7:**
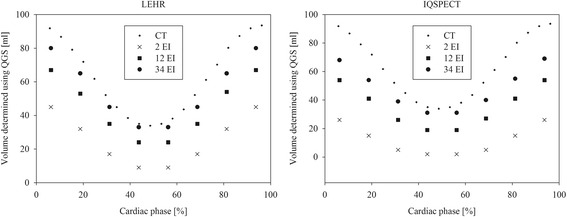
Cardiac phantom volume estimates for LEHR and IQ-SPECT protocols compared with corresponding CT measurements (*small dot*) with phantom EF setting 65%. The effect of the number of reconstruction iterations (EI) is shown with different markers (*cross* = 2, *square* = 12 and *large dot* = 34)

The best EF estimates and the associated number of EIs, EDVs and ESVs are shown in Table 2. Percentage difference (ΔEDV and ΔESV) between SPECT protocol and corresponding CT volumes are also shown. With the right choice of EIs both protocols produced EF values within one percentage point of the EF_True_. However, the absolute volume estimates were approximately 40 and 20% lower for IQ-SPECT and LEHR, respectively, when compared to ESV_True_ and EDV_True_. IQ-SPECT required a higher number of EIs to reach the EF_True_ values, possibly due to a smaller number of projections and a more complex reconstruction process. The slower convergence of the IQ-SPECT reconstruction is also shown in Fig. 7, where the cardiac cycle volumes of the phantom (phantom EF setting 65%) are compared with the corresponding CT data using different number of equivalent iterations.

**Table 2:** The best EF estimates of IQ-SPECT and LEHR imaging protocols and corresponding EI, EDV and ESV values analysed using QGS and 4DM software

QGS												
Phantom EF setting	IQ-SPECT						LEHR					
	EI	EF	EDV [ml]	ΔEDV [%]	ESV [ml]	ΔESV [%]	EI	EF	EDV [ml]	ΔEDV [%]	ESV [ml]	ΔESV [%]
45	21	45	38	−33	21	−36	12	44	44	−23	24	−26
55	17	53	42	−43	18	−44	12	54	54	−26	25	−23
65	16	65	54	−42	19	−41	12	65	67	−28	24	−26
70	16	70	63	−43	19	−41	14	69	80	−28	24	−26
4DM												
Phantom EF setting	IQ-SPECT						LEHR					
	EI	EF	EDV [ml]	ΔEDV [%]	ESV [ml]	ΔESV [%]	EI	EF	EDV [ml]	ΔEDV [%]	ESV [ml]	ΔESV [%]
45	21	45	38	−33	21	−35	30	45	55	−3	30	−7
55	16	56	42	−43	18	−44	20	58	54	−26	26	−20
65	18	67	54	−42	18	−44	16	65	74	−21	26	−20
70	22	70	57	−48	17	−47	10	70	74	−33	22	−32

The percentage differences, ΔESV and ΔEDV, to CT volumes are also shown

## Discussion

EF and LV volume estimates are clinically important parameters, but accurate volume determination is a non-trivial task prone to errors with any clinically available imaging modality [19]. Using gated MPI, the overall perfusion, EF and LV volumes can be estimated with a single imaging session simultaneously and the results are highly reproducible and in good agreement with other imaging techniques [20–23]. However, conventional MPI is time consuming for both the patient and for the imaging facility. Thus, there is a need for faster MPI acquisition techniques, both non-gated (for perfusion) and gated (LV function). IQ-SPECT presents an innovative combination of improved reconstruction methods and collimator design that increases system sensitivity at the centre of the cardio-centric orbit allowing reduced acquisition time or injected activity. Several groups around the world have compared IQ-SPECT with other nuclear medicine imaging techniques and protocols, although studies show conflicting results (see Background).

In this study, we have validated the volumes of a novel dynamic heart phantom using gated CT imaging and subsequently used the phantom to evaluate the accuracy of the EF estimates of two clinically used, IQ-SPECT and LEHR, MPI protocols. The CT results showed that phantom measurements were repeatable and accurate. Based on the measured EF accuracy (within 0.6% of the set values), consistency between different EF 65% acquisitions (within 0.2%), good correspondence to the values reported by the manufacturer and an acceptable level of uncertainty in the estimated absolute volumes, we conclude that the CT EF measurements are accurate enough to be treated as references for SPECT analyses.

Using the validated dynamic phantom, we showed that the EF and LV volume results using clinical IQ-SPECT and LEHR protocols are highly dependent on the number of reconstruction iterations (Fig. 5a, b). Both methods could accurately estimate the EF of the phantom, the error being one percentage point or less (Table 2). The absolute LV volumes were systematically underestimated more with IQ-SPECT than with LEHR (Fig. 6a, b). The LV volume calculations could be improved by using a higher number of iterations but that, in turn, would lead to increasingly underestimated EF values. These results imply that reporting the reconstruction parameters is of great importance, especially when comparing different MPI protocols. These findings can partly explain the inconsistency of the previously published results discussed in the Introduction.

The IQ-SPECT volumes were found to be systematically lower than the LEHR volumes. We presume that the underestimation of the volumes is mostly due to the limited number of views in the IQ-SPECT protocol and the LV volume estimates could be improved significantly if the number of views was increased. This hypothesis is supported by results from Horiguchi et al. [14], who showed that increasing the number of views from 17 to 36 in a ^201^Tl IQ-SPECT protocol results in more equivalent images to those acquired using conventional SPECT. We also hypothesize that increasing the number of views could rectify the deformed phantom shape in the IQ-SPECT (Fig. 4). Acquisition protocol optimisation is a very interesting topic and is currently under investigation by our group.

In this study, we did not use any background activity around the heart phantom since we aimed to omit confounding factors when studying IQ-SPECT and LEHR performance on myocardium phantom volume estimation. Our future studies, including imaging protocol optimisation, will include this omission. Attenuation correction was not used due to reconstruction software limitations for gated data. Further study is needed to determine if including attenuation correction in the reconstruction would improve the volume estimates as well as produce more faithful perfusion representation especially for gated IQ-SPECT.

Another issue that separates the phantom measurements from a clinical setting arises from analysis software used to analyse the gated SPECT images. The automatic myocardium delineation process in the QGS and 4DM software has an inherent anatomic model that may give different EF and LV volume results for a symmetrical phantom compared to a real human heart. All automated delineations were successfully processed without manual adjustment, and no clear faults in the delineations were not visible. We believe that the software effect on the results is small and, if present, is systematic and affects both LEHR and IQ-SPECT protocols equally.

In this study, all SPECT acquisitions were carried out using eight gating bins as per our standard clinical imaging protocol. The number of the bins affects the volume definition, and it has been shown that using eight bins underestimates LVEF by approximately 4% compared to 16 bins, primarily due to a poorer temporal resolution [20]. Fortunately, the underestimation is quite uniform over a wide range of LVEFs and can be taken into account when interpreting the LVEF values. In patient data, the use of 16 bins gating is usually limited by low count statistics of the SPECT projections that appears as poor image quality.

As IQ-SPECT has a high sensitivity, it should be possible to overcome the low count statistics and improve the volume assessment accuracy. However, the optimal acquisition protocol has still to be determined. Routinely, low statistics and noise in images are compensated by using post-reconstruction filters. Filtering helps to reduce noise but at the same time blurs the images to some extent. This evidently affects the SPECT volume and defect determination. Blurring caused by filtering has a similar effect on the LV volume and EF values as reducing the number of iterations has. From Fig. 5, we can see that the images with low number of EIs are blurred and that appears as underestimations of the LV volumes (Fig. 7) and an overestimation of the EF (Fig. 6). OSEM-based reconstruction algorithms tend to create grainy images if noise is present, and the number of iterations is increased. Because in our study a high activity concentration was used, this effect was not prominent and therefore no post-filtering was applied.

## Conclusions

We validated the volumes of a dynamic heart phantom using gated CT imaging. With the validated phantom, we showed that a clinical routine IQ-SPECT protocol produces systematically smaller LV volumes than a LEHR protocol. Both protocols could achieve accurate EF estimates, but the EF values were very dependent on the reconstruction parameters, which should be taken into account when comparing different imaging protocols. The optimal acquisition protocol for MPI with the IQ-SPECT warrants further study.
